# Intermittent hypoxia preconditioning can attenuate acute hypoxic injury after a sustained normobaric hypoxic exposure: A randomized clinical trial

**DOI:** 10.1111/cns.14662

**Published:** 2024-03-13

**Authors:** Yuan Wang, Qihan Zhang, Qingfeng Ma, Qing Wang, Dan Huang, Xunming Ji

**Affiliations:** ^1^ Department of Neurology, Xuanwu Hospital Capital Medical University Beijing China; ^2^ Development Coordination Office Beijing Xiaotangshan Hospital Beijing China; ^3^ Department of Neurosurgery, Xuanwu Hospital Capital Medical University Beijing China

**Keywords:** acute hypoxic injury, brain injury, intermittent hypoxia, intracranial pressure, preconditioning

## Abstract

**Background:**

Intermittent hypoxia (IH) is emerging as a cost‐effective nonpharmacological method for vital organ protection. We aimed to assess the effects of a short‐term moderate intermittent hypoxia preconditioning protocol (four cycles of 13% hypoxia lasting for 10 min with 5‐min normoxia intervals) on acute hypoxic injury induced by sustained hypoxic exposure (oxygen concentration of 11.8% for 6 h).

**Methods:**

One hundred healthy volunteers were recruited and randomized to the IH group and the control group to receive IH or sham‐IH preconditioning for 5 days, respectively, and then were sent to a hypoxic chamber for simulated acute high‐altitude exposure (4500 m).

**Results:**

The overall incidence of acute mountain sickness was 27% (27/100), with 14% (7/50) in the IH group and 40% (20/50) in the control group (*p* = 0.003). After 6‐h simulated high‐altitude exposure, the mean Lake Louise Score was lower in the IH group as compared to controls (1.30 ± 1.27 vs. 2.04 ± 1.89, *p* = 0.024). Mean peripheral oxygen saturations (SpO_2_) and intracranial pressure (ICP) measures after acute hypoxic exposure exhibited significant differences, with the IH group showing significantly greater SpO_2_ values (85.47 ± 5.14 vs. 83.10 ± 5.15%, *p* = 0.026) and lower ICP levels than the control group (115.59 ± 32.15 vs. 130.36 ± 33.83 mmH_2_O, *p* = 0.028). IH preconditioning also showed greater effects on serum protein gene product 9.5 (3.89 vs. 29.16 pg/mL; *p* = 0.048) and C‐reactive protein (−0.28 vs. 0.41 mg/L; *p* = 0.023).

**Conclusion:**

The short‐term moderate IH improved the tolerance to hypoxia and exerted protection against acute hypoxic injury induced by exposure to sustained normobaric hypoxia, which provided a novel method and randomized controlled trial evidence to develop treatments for hypoxia‐related disease.

## Graphical abstract

In this trial, an intermittent hypoxia preconditioning protocol of four cycles of 13% hypoxia lasting for 10 min with 5‐min normoxia intervals was found to exert protective effects on acute hypoxic injury. Intermittent hypoxia preconditioning has the potential to reduce the occurrence and severity of acute mountain sickness in hypoxic condition, improve peripheral oxygen saturation, reserve intracranial pressure, and lessen elevation of inflammatory and brain injury biomarkers. The figure of Graphical Abstract was created with Biorender.com.

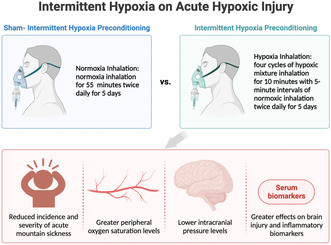

## 1 INTRODUCTION

Oxygen is critical for mammalian survival, and regulating oxygen levels within a limited range is necessary to optimize biological functions.^1^ Although oxygen deficiency is a common factor both in hypoxia and ischemia, ischemia is a hypoxic episode characterized by insufficient nutrient supply due to decreased perfusion.^2^ While respiratory hypoxia involves decreased oxygen concentration in the tissue without affecting blood flow and nutrient supply.^3^ Environmental hypoxia, defined as decreased partial pressure of oxygen in inspired air, is common in aerospace activities, deep sea diving, mountain climbing, and plateau traveling, and it usually increases the probability of body or organ damage.^4^ Decompensation for acute hypoxic conditions, such as rapid ascent to high altitude, may result in acute hypoxic injury and nonspecific symptoms, including headache, nausea, vomiting, and fatigue. This is a clinical syndrome termed altitude illness, ranging from mild acute mountain sickness (AMS) to more serious high‐altitude cerebral edema or high‐altitude pulmonary edema.^5^ About half of unacclimatized individuals develop AMS of varying severity within hours to days after rapid ascent to altitudes greater than 2500 m. If AMS symptoms are ignored, they may develop into potentially fatal high‐altitude cerebral edema or high‐altitude pulmonary edema.^6^

Intermittent hypoxia (IH), defined as alternating bouts of breathing hypoxic and normoxic air, was considered a central feature of obstructive sleep apnea in the past.^7^ As a double‐edged sword, tissue and organs engage in producing biphasic effects in response to hypoxia based on IH protocols. The frequency, duration, and degree of IH episodes are pivotal factors in their physiological impact.^8^ Depending on complex protective mechanisms, IH counteracts the damage of hypoxia to organisms and exerts multiple beneficial effects on the cardiovascular, respiratory, nervous system, blood system, and others.^9^ Previous studies indicated that the protocols consisting of modest hypoxia, low cycle numbers, and short duration appear to produce beneficial effects without pathology.^8^

Nowadays, preventative strategies for AMS mainly include the oral administration of acetazolamide, dexamethasone, and a slow ascent rate at high altitudes, which still cannot make people adapt to acute hypoxia sufficiently.^10^ Intermittent hypoxia preconditioning might be a simple, safe, and effective nonpharmacological method to induce acclimatization and prevent acute hypoxic injury.^8^

Based on previous studies, we further hypothesized that a short‐term moderate normobaric IH protocol, characterized by 4 hypoxic periods, each lasting 10 min with a 5‐min normoxic interval, is effective in improving the tolerance to hypoxia and ameliorating acute hypoxic injury.

## 2 METHODS AND MATERIALS

### 2.1 Subjects

A total of 100 healthy subjects residing at low altitudes were recruited to participate in the study and randomized to the control group and IH group at a 1:1 ratio according to a computer‐generated randomization table from March 1, 2023, to September 1, 2023. A comprehensive and detailed physical examination and clinical evaluation were performed before enrollment to single out the ones who met the inclusion criteria and who needed to be excluded. The inclusion criteria included: (1) healthy lowlanders of both sexes aged from 18 to 45 years old with body mass index between 19 and 24.9 kg/m^2^; (2) resting peripheral oxygen saturation (SpO_2_) of more than 90%, cerebral oxygen saturation between 58% and 82%, heart rate between 60 and 100 beats/min, and blood pressure within the normal range (90–130/60–80 mmHg). Exclusion criteria were a history of respiratory, cardiovascular, neurological, and hematological diseases, including chronic respiratory diseases, cerebrovascular diseases, and anemia. Subjects under the categories of menstruating, pregnant, lactating, smoking, alcohol intake habits, and long‐living at altitudes >1200 m, as well as high‐altitude exposure in the last 6 months, were also excluded. Subjects were asked to wean off caffeine, alcohol, functional beverages, and strenuous exercise throughout the testing period. All the subjects were apprised of the scope of the experiment and provided informed written consent. This study was conducted in accordance with the reporting guidelines of Consolidated Standards of Reporting Trials,^11^ and had been approved by the Ethics Committee of Xuanwu Hospital, Capital Medical University, and registered on the site ClinialTrials.gov (https://clinicaltrials.gov; NCT05733338).

### 2.2 Overall design

**FIGURE 1:**
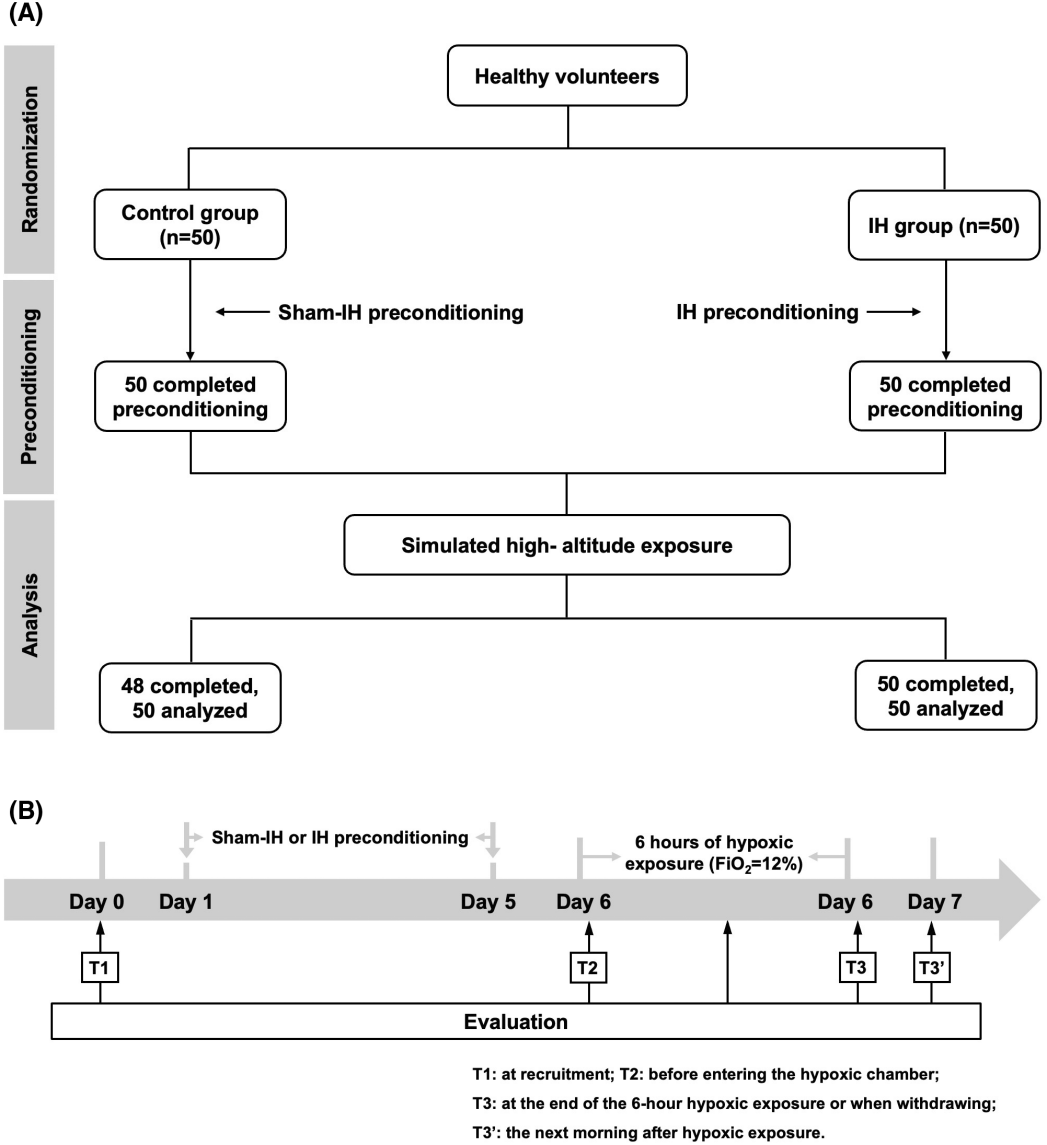
Overall experimental design. (A) Flow diagram illustrating the progress of the study. (B) Experimental design. FiO_2_, fraction of inspired oxygen; IH, intermittent hypoxia. T1: at recruitment; T2: before entering the hypoxic chamber; T3: at the end of the 6‐h hypoxic exposure or when withdrawing; T3′: the next morning after hypoxic exposure.

This study was performed as a double‐blind, placebo‐controlled design, in which subjects were randomized to receive either IH preconditioning (IH group, *n* = 50) or sham‐IH preconditioning (control group, *n* = 50) for 5 days prior to the sustained hypoxic exposure on the 6th day (Figure 1). Neither the subjects nor the investigators assessing outcomes were aware of the intervention assignments until the end of the trial. The primary outcome was the occurrence and severity of AMS, as assessed after 6‐h acute hypoxic exposure. Subjects' blood pressure, heart rate, SpO_2_, as well as intracranial pressure (ICP) were recorded. Blood biomarkers were tested. The safety endpoint was the incidence of severe or intolerable discomfort and termination conditions.

### 2.3 Intermittent hypoxia preconditioning protocol

Subjects in the IH group were trained with the IH protocol, which is, a 10‐min 13% fraction of inspired oxygen (FiO_2_) alternated by 5 min 21% FiO_2_ for four sessions, while the control group received 21% FiO_2_ sustaining 55 min. The preconditioning was performed twice a day for 5 consecutive days. The optimal IH protocol that elicits protective effects has not been identified. Emerging evidence suggested that IH protocols characterized by mild to moderate hypoxia (FiO_2_ = 9%–16%), short in duration (3–10 min), and 3–15 cycles/day generally yield beneficial outcomes without pathological ones. The IH protocol we chose was in line with these principles.^12^

Intermittent hypoxia was carried out by inhaling hypoxic gas mixtures with reduced FiO_2_ under a normobaric atmosphere conducted by a hypoxicator.^13^ The hypoxic apparatus was designed to work by compressing air and separating the oxygen from the nitrogen, then mixing the two at a set level to achieve the targeted oxygen saturation, which was transmitted to the subject through a face mask. Following each hypoxic episode, subjects were instructed to remove the masks during the normoxic interval. The whole IH intervention was performed by well‐trained physicians.

### 2.4 Acute hypoxic exposure

Simulated high‐altitude exposure was conducted at Beijing Highland Conditioning Medical Center. An acute hypoxic environment was attained by the normobaric hypoxic chamber of the center.^14^ An altitude of 4500 m was simulated, and the total pressure was one atmospheric pressure (101.3 kPa) at sea level, with an oxygen proportion of 11.8% and a nitrogen of 88.2%. Subjects were sent to this normobaric hypoxic chamber for 6 h for acute hypoxic exposure on the following day after finishing the 5‐day preconditioning process. Symptoms of AMS usually occur within 6–12 h following arrival at high altitude above 3000–4000 m. However, the risk of high‐altitude cerebral edema and high‐altitude pulmonary edema increases significantly at altitudes higher than 4500–5000 m.^6^ As a result, the 6‐h simulated altitude of 4500 m (with an oxygen proportion of 11.8%) was adopted. The hypoxic chamber can be opened at any time and is equipped with medical personnel and emergency medicine to deal with sudden severe intolerant symptoms.

### 2.5 Assessment

#### 2.5.1 Assessment of clinical symptoms

The Lake Louise Score (LLS) system was introduced to assess the incidence and severity of AMS. It is a self‐reporting questionnaire including four symptoms: headache, gastrointestinal symptoms, fatigue and/or weakness, dizziness/light‐headedness, rating from 0 (not present) to 3 (severe). Subjects were considered to suffering from AMS with a total score ≥3 and at least one point from headache.^15^ After the 6‐h hypoxic exposure or when withdrawing from the hypoxic chamber due to the development of intolerable symptoms, this scale was delivered to the subjects concerning their subjective symptoms and completed by themselves, with oral instructions by the investigators. Additionally, as an essential element of human well‐being that is independent of symptoms of AMS, sleep could be disturbed because of periodic breathing in hypoxia, so the study also evaluated sleep quality by the Insomnia Severity Index.^16^ The questionnaire was administered to the subjects at three timepoints: at recruitment (T1), before entering the hypoxic chamber (T2), and the next morning after hypoxic exposure (T3′).

#### 2.5.2 Assessment of physiological parameters

Measurements of physiological parameters were performed in quiet rooms, and participants were tested in a seated position after at least 5‐min rest. The peripheral oxygen saturation, heart rate, and blood pressure of each subject were monitored by multiparameter patient monitor apparatus (ePM12, Shenzhen Mindray Bio‐Medical Electronics Co., Ltd., Shenzhen, China), and was acquired at the following time points: at recruitment (T1), before entering the hypoxic chamber (T2), every 1 h during the 6‐h hypoxic exposure period (five times), and at the end of the 6‐h hypoxic exposure or when withdrawing (T3).

#### 2.5.3 Assessment of intracranial pressure

Intracranial pressure was noninvasively measured by intracranial pressure noninvasive comprehensive detection analyzer (JYH_ICP_1B_D, Chongqing Zhongli Medical Equipment Co., Ltd., Chongqing, China) at recruitment (T1), before entering the hypoxic chamber (T2), and at the end of the 6‐h hypoxic exposure or when withdrawing (T3).

#### 2.5.4 Safety endpoints

The 6‐h hypoxic exposure should be terminated if subjects developed intolerable discomfort (subjective termination conditions) or evaluated by the physician that were not suitable to continue (objective termination conditions). Intolerable discomfort is defined as headache, gastrointestinal symptoms, fatigue and/or weakness, dizziness/light‐headedness that is so severe that the person is unable to live or work normally (refer to the highest score of each symptom of LLS), or severe ataxia, shortness of breath, dyspnea, etc. Objective termination criteria, as judged by the physician, included any of the following indicators: SpO_2_ ≤ 80%; blood pressure ≥160/100 mmHg, heart rate ≥100 beats/min or increase ≥10% from the baseline, any electrocardiography signs of myocardial ischemia or malignant arrhythmia, respiration rate ≥40 breaths/min. The occurrence of high‐altitude cerebral edema or high‐altitude pulmonary edema was also recorded, which manifested as symptoms of wobbly gait, confusion, decreased consciousness, excessive exertional dyspnea, mild cough, chest tightness, and reduced exercise performance.^6^ For subjects who met the criteria of termination, they were sent to the medical care room to accept acetazolamide (250 mg every 12 h), dexamethasone (4 mg every 6 h) and/or oxygen inhaling (2 L/min), and pain relievers (paracetamol, 500–1000 mg) or antiemetics (metoclopramide, 10 mg) were administered when necessary. If the vital signs were stable and the symptoms were significantly improved, the subjects could be granted leave.

### 2.6 Blood test

This study monitored protein gene product 9.5 (PGP 9.5), glial fibrillary acidic protein (GFAP), calcium channel binding protein S100 subunit beta (S100β) as brain injury indicators, and interleukin‐6 and C‐reactive protein (CRP) as inflammatory indicators. Two milliliter resting venous blood samples from an arm vein were taken each time at three time points: at recruitment (T1), before entering the hypoxic chamber (T2), and at the end of the 6‐h hypoxic exposure or when withdrawing (T3). The serum was separated by centrifugation for 10 min at 2000 *g* and stored in −80°C freezers. To reduce the inter‐assay error and measurement error, the biomarkers for all participants were measured simultaneously. The detection of these indicators was performed automatically by chemiluminescence using the 80A automated chemiluminescent immunoassay analyzing system (Sophonix Co., Ltd., Beijing, China). All serological assays were carried out in strict accordance with the manufacturer's instructions.

### 2.7 Statistical analysis

The statistical analyses were performed using IBM SPSS Software 26.0 (IBM, Armonk, NY, USA). Quantitative variables were presented as mean ± standard deviation if a normal distribution was established, or as median (interquartile range) for data with a skewed distribution. All data were tested for normality through the Shapiro‐Wilk test. Data that did not exhibit a normal distribution were analyzed via a nonparametric equivalent. For continuous variables that fit a normal distribution, unpaired *t*‐tests were used to evaluate differences between groups at different time points, and student *t*‐tests were used for within‐group comparisons. Qualitative and not normally distributed data were processed through nonparametric statistics (Mann‐Whitney *U* test and Wilcoxon). A chi‐square test was used for comparing categorical variables between groups. Spearman's correlation analyses were applied to assess the relationship between the SpO_2_ and LLS after acute hypoxic exposure. Statistical significance was set at *p* values < 0.05. The sample size was calculated by setting type I error at two‐sided 0.05 and type II error at 0.20. Based on previous studies showing that intermittent hypoxia can alleviate AMS, which were reduced from approximately 50% to 10%, a clinically acceptable margin was assumed to be a difference of 12% between the two groups.^17^ The sample size required for the study was calculated to be 40 per group, and considering a rate of 20% to drop out or withdraw, 50 cases are needed in each group, for a total of 100 volunteers to be randomized.

## 3 RESULTS

Of the 100 subjects included in this study, 51 were male. The mean age and body mass index of all subjects were 35.46 ± 6.16 years and 23.89 ± 3.18 kg/m^2^, respectively. All subjects lived at an altitude of less than 100 m for a long time. All subjects well tolerated the preconditioning program without adverse effects. Ninety‐eight subjects completed the 6‐h hypoxic exposures, whereas two subjects in the control group terminated the trial because of severe and intolerable discomfort, the data of whom were collected at the time of withdrawing. The rate of termination showed no statistical difference between the two groups (0% vs. 4%, *χ* ^2^ = 0.510, *p* = 0.475). No severe complications such as high‐altitude cerebral edema, or high‐altitude pulmonary edema occurred in the simulated high‐altitude. Demographical data were presented in Table S1.

### 3.1 Intermittent hypoxia relieves symptoms related to acute hypoxia

**FIGURE 2:**
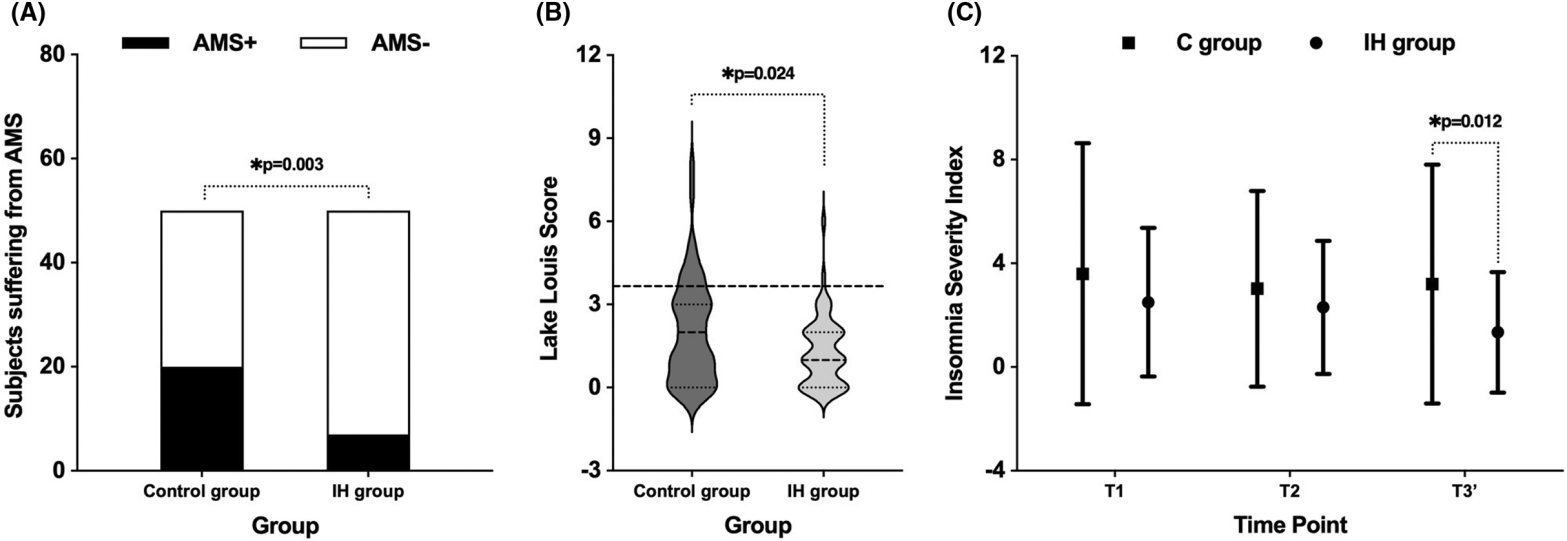
The incidence and severity of AMS and sleep quality of two groups. (A, B) The occurrence and severity of AMS in the IH group were significantly lower than that in control group after the 6‐h simulated high‐altitude exposure. (C) The comparison of sleep quality of two groups in three evaluated time points showed significantly lower score in the IH group than control group in T3′. T1: at recruitment; T2: before entering the hypoxic chamber; T3′: the next morning after hypoxic exposure. *p < 0.05.

In total, 14% (7/50) of the IH group and 40% (20/50) of the control group developed AMS after the 6‐h hypoxic exposure [95% confidence interval 0.09–0.43, *χ* ^2^ = 8.574, *p* = 0.003] (Figure 2A). The absolute risk of AMS decreased by 26% after IH preconditioning. Compared with the control group, subjects in the IH group reported lower LLS (1.30 ± 1.27 vs. 2.04 ± 1.89, *p* = 0.024) (Figure 2B).

There was no statistical difference of Insomnia Severity Index scores between the two groups at T1 and T2. At T3′, mean score in the IH group was significantly lower than the control group (1.34 ± 2.33 vs. 3.20 ± 4.61, *p* = 0.012) (Figure 2C).

### 3.2 Intermittent hypoxia improves the tolerance to acute hypoxia

**FIGURE 3:**
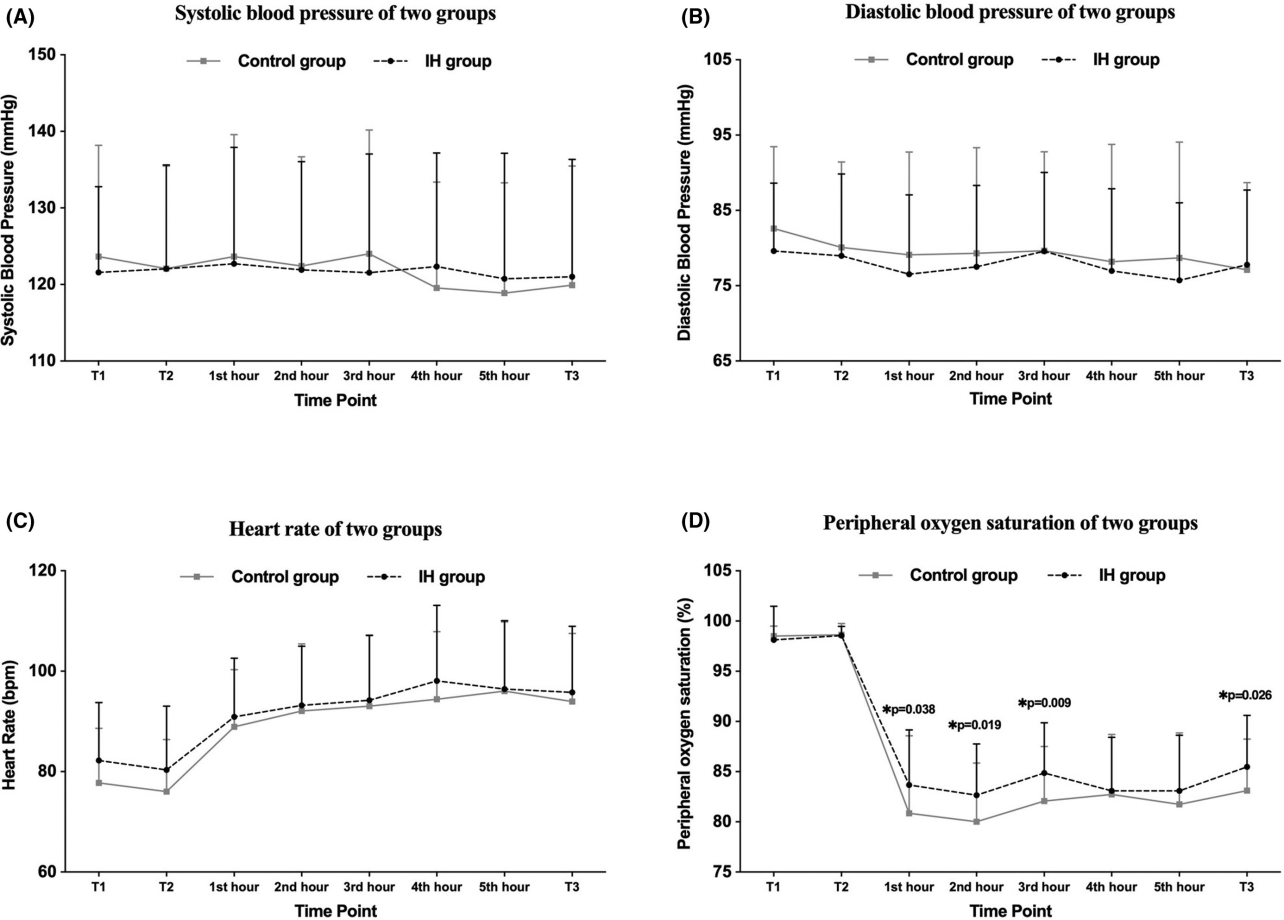
Physiological parameters were recorded at eight time points. (A–D) Fluctuation of systolic blood pressure, diastolic blood pressure, heart rate, and peripheral oxygen saturation. T1: at recruitment; T2: before entering the hypoxic chamber; T3: at the end of the 6‐h hypoxic exposure or when withdrawing. *p* values represent the comparison between the two groups at the specific time point. *p < 0.05.

**FIGURE 4:**
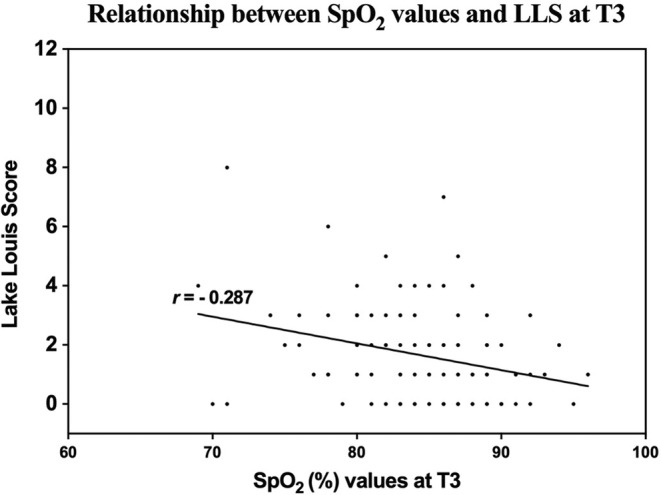
Correlation between SpO_2_ and AMS severity. There was a significant negative correlation between SpO_2_ and AMS severity after acute simulated high‐altitude exposure.

Subjects' blood pressure, heart rate, and SpO_2_ values were obtained across all eight‐time assessments (Table S2) and the fluctuation of which were shown in Figure 3. Systolic blood pressure, diastolic blood pressure, and heart rate did not differ significantly between groups at T1, T2, and each evaluated time point during the 6‐h hypoxic environment exposure (*p* > 0.05). There was no statistical difference in SpO_2_ between the two groups at T1 and T2. After 1 h exposure to hypoxia, SpO_2_ in the IH group and the control group decreased to 85.47 ± 5.14% and 83.10 ± 5.15% respectively, which was with significant difference (*p* = 0.038). Similarly, significant differences in SpO_2_ between the two groups were also found at the 2nd hour, 3rd hour, and T3 of hypoxic exposure. Compared to the IH group, SpO_2_ after the 6‐h hypoxic exposure showed a greater decline from T1 in the control group (−13.10 ± 5.11 vs. −15.60 ± 5.14%, *p* = 0.018). The subjects were then divided into those who suffered from AMS (AMS+) and those who stayed well (AMS−). Mean SpO_2_ values were significantly lower in AMS+ compared with AMS− (81.78 ± 5.29 vs. 85.14 ± 5.14%, *p* = 0.007). Additionally, to explore the interrelationship between SpO_2_ and AMS severity, we performed a correlation analysis, and found that LLS score was negatively correlated with SpO_2_ values at T3 (*r* = −0.287, *p* = 0.004, Figure 4).

### 3.3 Intermittent hypoxia alleviates intracranial hypertension induced by acute hypoxia

**FIGURE 5:**
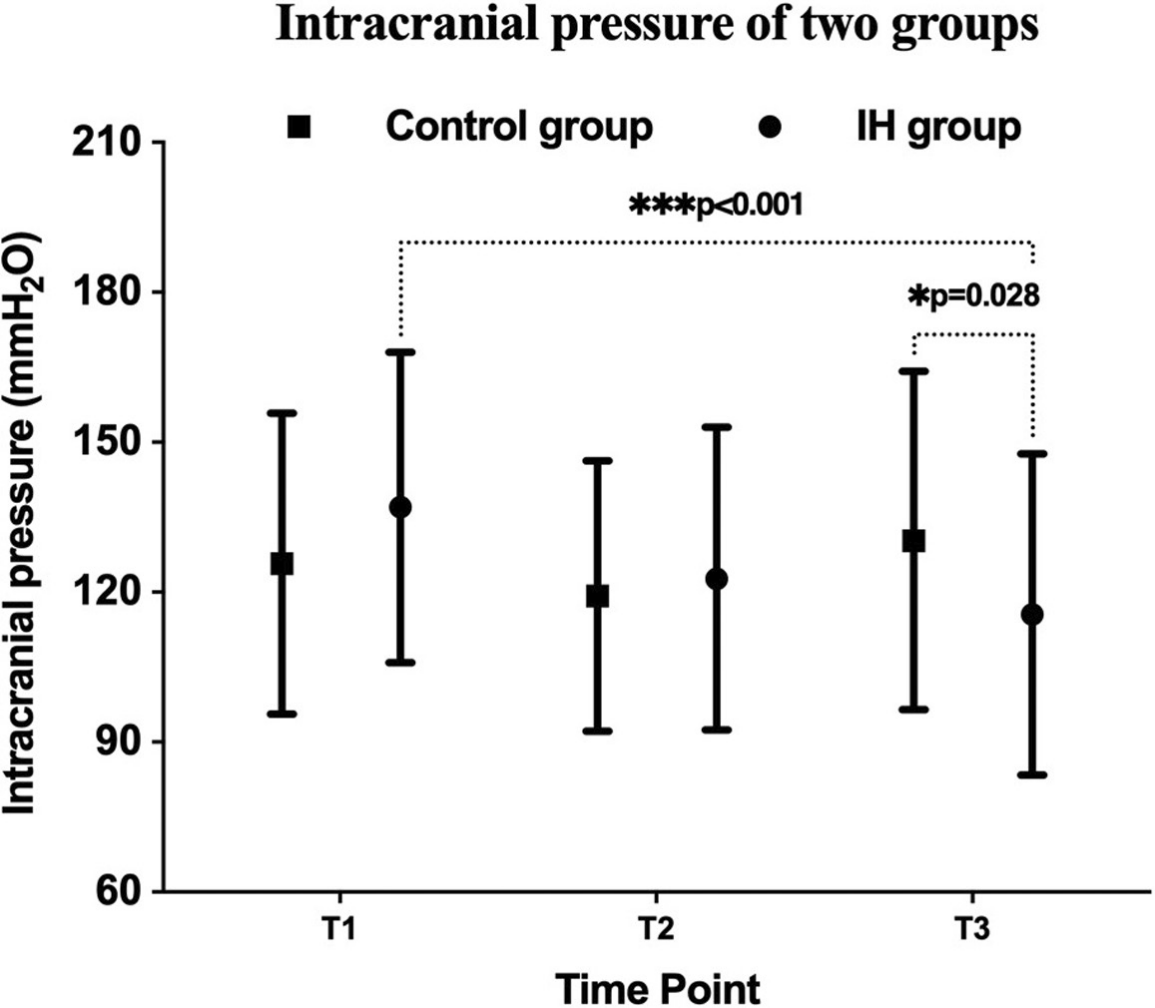
Intracranial pressure of two groups in three evaluated time points. The values of intracranial pressure showed statistically significant differences between the two groups at T3, and within the IH group between T1 and T3. T1: at recruitment; T2: before entering the hypoxic chamber; T3: at the end of the 6‐h hypoxic exposure or when withdrawing. *p < 0.05; ***p < 0.001.

There were no significant differences in ICP between groups at T1 (137.00 ± 31.05 vs. 125.75 ± 30.10 mmH_2_O, *p* = 0.069) and T2 (122.72 ± 30.28 vs. 119.27 ± 27.02 mmH_2_O, *p* = 0.549). After the IH or sham‐IH preconditioning, ICP decreased by 14.28 ± 38.13 mmH_2_O and 6.48 ± 26.13 mmH_2_O, respectively (*p* = 0.188). After the 6‐h simulated high‐altitude hypoxic exposure, ICP in the IH group was lower than the control group (115.59 ± 32.15 vs. 130.36 ± 33.83 mmH_2_O, *p* = 0.028) (Figure 5). The changes in ICP after the 6‐h hypoxic exposure from the baseline between the two groups were also significant (−21.41 ± 38.90 vs. 4.60 ± 41.47 mmH_2_O, *p* = 0.002).

### 3.4 Intermittent hypoxia attenuates brain injury and inflammation caused by acute hypoxia

**FIGURE 6:**
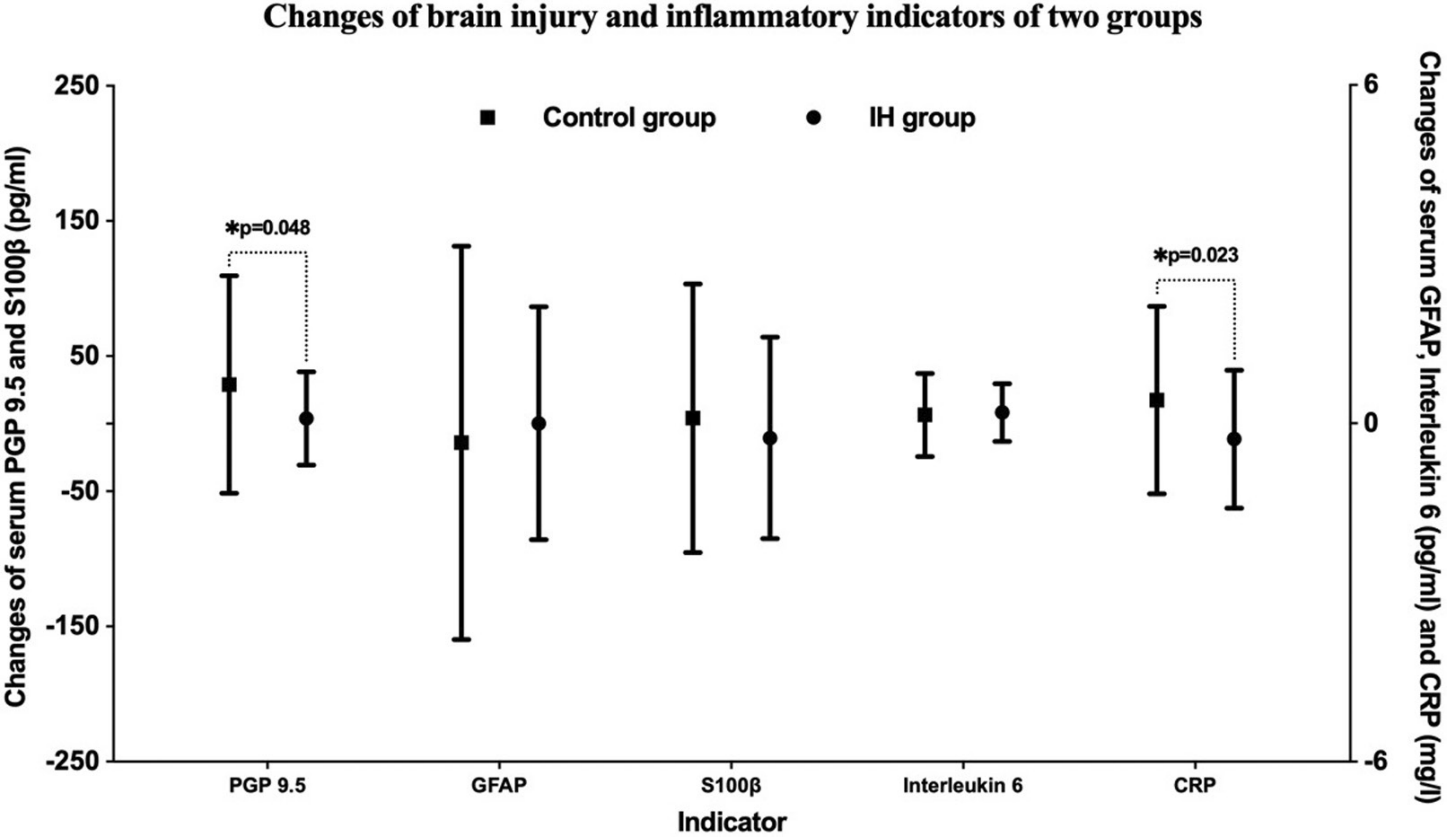
Changes of brain injury and inflammatory indicators from baseline to the 6‐h hypoxic exposure. CRP, C‐reactive protein; GFAP, glial fibrillary acidic protein; PGP 9.5, protein gene product 9.5; S100β, calcium channel binding protein S100 subunit beta. *p < 0.05.

No significant differences between groups at T1 and T2 in these biomarkers' levels were found, whereas PGP 9.5 values showed a significant difference between groups at T3 (92.63 ± 35.88 vs. 120.71 ± 89.74 pg/mL, *p* = 0.047) (Table S3). The mean change from baseline to the end of the 6‐h hypoxic exposure in PGP 9.5 was 3.89 pg/mL for the IH group and 29.16 pg/mL for the control group (*p* = 0.048). The level of CRP in the IH group reduced significantly after the 6‐h hypoxic exposure relative to the control group (−0.28 vs. 0.41 mg/L, *p* = 0.023) (Figure 6).

## 4 DISCUSSION

Intermittent hypoxia refers to periodic exposure to hypoxia, interspersed by normoxia. Various IH protocols mainly differ in the degree of hypoxic stimulation, duration, and frequency of hypoxic episodes. IH protocols of short‐term, mild to moderate hypoxia can increase the resistance to hypoxic injury, which are being used as a nonpharmacological strategy to manifest cardio and neuroprotection, improve metabolic status, enhance athletic performance, pre‐acclimatization to high‐altitude, and other pathological conditions.^8^ These IH protocols are substantially different from obstructive sleep apnea, which is a potential risk factor associated with systemic diseases, metabolic dysfunction, and cognitive impairment.

In this study, we evaluated the effects of IH on acute hypoxic injury through exposure to a simulated altitude of 4500 m. The present results suggest that IH can effectively relieve symptoms induced by acute hypoxia, improve tolerance to hypoxia, and reduce brain injury and inflammation. The study indicated that the short‐term moderate IH supports passive acclimatization to hypoxia, which provided a perspective for developing treatment for hypoxia‐related disease.

Generally, people develop various clinical syndromes within 6–12 h of acute hypoxic exposure at an altitude of more than 2500 m, and the incidence of AMS varies between 40% and 90%, which is related to the rate of ascent, altitude reached, and individual susceptibility.^6^, ^18^ In the present study, the AMS incidence was reduced by 26% in the IH group relative to the control group after exposure to a simulated altitude of 4500 m. Compared with the control group, subjects in the IH group showed a lower LLS, which manifested the severity of AMS. Insomnia is another common complaint. We also assessed the sleep quality after the 6‐h hypoxic exposure, and the mean score of the Insomnia Severity Index in the IH group was also lower than that in the control group.

Hypoxia plays a central role in the pathogenesis of AMS. Hypoxemia happened to subjects when they are exposed to hypoxic environments and is exaggerated in those who develop AMS.^19^ In the present study, we found that the SpO_2_ level in subjects who suffered from AMS was much lower than those who stayed well. And there was a negative relationship between the SpO_2_ values and LLSs after 6 h of hypoxic exposure. Repeated IH exposures were found to induce hypoxia‐responsive genes, enhance systemic oxygen delivery, and improve the tolerance to hypoxia.^14^, ^20^ Compared to the control group, IH‐trained subjects possessed approximately 2% higher SpO_2_ values after acute hypoxic exposure, with a lower AMS incidence, lower LLSs, and lower Insomnia Severity Index scores. These results suggested that IH preconditioning improves oxygen saturation in an acute hypoxic environment and facilitates hypoxia acclimatization, which is in line with previous studies.^7^, ^14^, ^21^, ^22^

The development of AMS results from complex physiological responses to hypoxia, such as inflammation, vasogenic edema, as well as anatomical factors.^23^ Increased ICP caused by cerebral edema and elevated cerebral perfusion is one of the major complications of acute hypoxic injury.^24^ Exposure to a hypoxic environment induces cerebral vasodilation, which can increase the cerebral blood volume and diminish cerebrovascular compliance, eventually leading to an increase in ICP.^25^ Furthermore, increased ICP and cerebral vasodilation may trigger activation of the trigeminal vascular system and cause headaches.^19^, ^26^ Our study found that IH preconditioning can remit ICP. Compared with the control group, the IH group showed a lower ICP after the hypoxic exposure. From baseline to the end of 6‐h acute hypoxic exposure, ICP increased by 4.60 mmH_2_O in the control group, whereas it decreased by 21.41 mmH_2_O in the IH group. Thus, IH preconditioning may be helpful in alleviating increased ICP related to acute hypoxic injury.

The brain relies on a constant and adequate supply of blood flow and oxygen delivery for its survival, which makes it sensitive to hypoxia. We used the pan‐neuronal marker PGP 9.5 and the glial markers S100β and GFAP as the cerebral damage‐related markers.^27^ A significant training difference favoring IH preconditioning was observed in the PGP 9.5 levels after the 6‐h hypoxic exposure, which was also found in the mean change of PGP 9.5 concentrations from baseline to the end of the 6‐h hypoxic exposure. PGP 9.5, a human neuron‐specific ubiquitin carboxyl‐terminal hydrolase, has been proposed as a biomarker of brain injury because of its high and specific expression in the nervous system.^28^ And researchers have found that PGP 9.5 levels were elevated in patients with hypoxic–ischemic brain injury, which may be attributed to injury to the neurovascular unit.^29^ Our study thus corroborates that levels of PGP 9.5 are elevated under acute hypoxic conditions and that this effect can be attenuated by IH preconditioning. Additionally, it has been reported that inflammation is also a key component in the physiological response to acute hypoxic stress.^30^ The release of inflammatory and angiogenic mediators initiated by hypoxia seems to disrupt the blood–brain barrier and promote vasogenic edema.^31^, ^32^ It has been reported that circulating inflammatory markers, most notably CRP, interleukin‐1β, and interleukin 6, are elevated in individuals who are acutely exposed to hypoxia or high altitude.^23^, ^33^ Persistent high levels of inflammation are associated with altitude diseases such as AMS,^34^ and inhibition of the inflammatory response has been reported to exert protective effects on AMS.^31^, ^35^ In our study, there was a significant reduction in PGP 9.5 and CRP levels in the IH group. Thus, IH can mitigate inflammation and attenuate brain damage induced by hypoxia, which may contribute to the protection of clinical manifestations.

Despite the beneficial outcomes of this study that have been described above, the potential risks associated with IH should not be neglected. Hypoxemia, which may occur during the conditioning process, could elicit uncomfortable symptoms like headaches, difficulty breathing, and increased heart rate. When the IH protocols are not appropriate, IH might increase arterial blood pressure and intracranial pressure, and impair cognitive function.^8^ In general, the main factors determining the beneficial or detrimental direction of IH are the hypoxic intensity and duration of exposure. As a result, the implementation of IH must be monitored to minimize these potential risks. In a previous study, we found that the IH protocol used in our trial did not cause unbearable discomfort or adverse events, which was well tolerated in healthy volunteers. During the IH intervention, subjects' blood pressures, heart rates, peripheral oxygen saturations, and cerebral oxygen saturations fluctuated within the normal range.^13^ In the present study, all participants completed the preconditioning program with good tolerance and no adverse effects.

This study has several limitations. First, the sample size of this study was still small. Second, our investigations have been solely performed in normobaric hypoxia, and the duration of hypoxic exposure was relatively short, and the physiological effects of hypobaric and normobaric hypoxia may differ. Third, we found the protective effects of IH but did not determine the underlying mechanisms. Fourth, despite including healthy subjects without a history of respiratory, cardiovascular, neurological, and hematological diseases, we did not completely eliminate confounding factors that might influence the outcomes, such as pulmonary function, arterial blood gas analysis, and hemoglobin levels. Finally, the current evidence does not establish the absolute safety of intermittent hypoxia, and the monitoring and evaluation system is still incomplete. Further investigations are needed to validate the beneficial effects of intermittent hypoxia and the underlying mechanisms, as well as to determine the appropriate dose to promote the effects while reducing potential risks.

## 5 CONCLUSION

This placebo‐controlled, double‐blinded study found that the specified short‐term IH preconditioning protocol (alternate exposure to hypoxia for 10 min and normoxia for 5 min for four cycles, twice a day for 5 days) exerts protective effects on acute hypoxic injury. The results indicated that IH preconditioning has the potential to not only reduce the occurrence of AMS but also alleviate AMS severity in simulated hypoxic condition, and these effects could be attributed to improved SpO_2_, reserved ICP, and reduced inflammation and brain damage. Thus, intermittent normobaric hypoxia preconditioning supports passive acclimatization to sustained hypoxia. Our study provides new methods and randomized controlled trial (RCT) evidence for the application of intermittent hypoxia on hypoxia‐related disease.

## FUNDING INFORMATION

This work was supported by National Key R&D Program of China (grant number 2022YFC3501005) to Wang Y. Ji XM was supported by the National Natural Science Foundation of China (grant number 82027802).

## CONFLICT OF INTEREST STATEMENT

The authors declare no conflicts of interest.

## PATIENT CONSENT STATEMENT

All the subjects provided informed written consent in this study.

## CLINICAL TRIAL REGISTRATION

This study has been registered on the site ClinialTrials.gov (https://clinicaltrials.gov), registration number: NCT05733338.

## Supporting information


Tables S1–S3

## Data Availability

The datasets supporting the conclusions of the current study are available from the corresponding author upon reasonable request.
