# Supplementary material for: Intermittent hypoxia preconditioning can attenuate acute hypoxic injury after a sustained normobaric hypoxic exposure: A randomized clinical trial
Source: CNS Neurosci Ther. 2024 Mar 13;30(3):e14662. doi: 10.1111/cns.14662 (PMC10934266; doi:10.1111/cns.14662)
Supplement: Supplementary file 1 — Tables S1–S3 [file CNS-30-e14662-s001.docx]

Supplement Table 1. Demographical data

|  | Control group (n=50) | IH group (n=50) | P |
| --- | --- | --- | --- |
| Age (years, mean±standard deviation) | 35.36±5.64 | 35.56±6.70 | 0.872 |
| Male (n, %) | 26 (52%) | 25 (50%) | 0.841 |
| Height (cm, mean±standard deviation) | 169.34±7.94 | 167.32±9.02 | 0.237 |
| Body mass (kg, mean±standard deviation) | 69.12±13.04 | 67.02±12.17 | 0.407 |
| Body mass index (kg/m^2^, mean±standard deviation) | 23.95±3.16 | 23.84±3.23 | 0.854 |

IH, intermittent hypoxia.

Supplement Table 2. Physiological parameters between groups

|  | Time points | Groups | | P |
| --- | --- | --- | --- | --- |
|  |  | Control group | IH group |  |
| Systolic blood pressure (mmHg) | T1 | 123.66±14.52 | 121.58±11.21 | 0.425 |
|  | T2 | 122.10±13.38 | 122.04±13.58 | 0.982 |
|  | 1^st^ hour | 123.66±15.94 | 122.70±15.22 | 0.759 |
|  | 2^nd^ hour | 122.41±14.28 | 121.92±14.13 | 0.865 |
|  | 3^rd^ hour | 124.02±16.17 | 121.54±15.52 | 0.438 |
|  | 4^th^ hour | 119.54±13.84 | 122.35±14.84 | 0.338 |
|  | 5^th^ hour | 118.90±14.38 | 120.76±16.41 | 0.555 |
|  | T3 | 119.92±15.57 | 121.02±15.34 | 0.723 |
| Diastolic blood pressure (mmHg) | T1 | 82.60±10.86 | 79.60±9.03 | 0.136 |
|  | T2 | 80.10±11.35 | 78.96±10.89 | 0.610 |
|  | 1^st^ hour | 79.12±13.63 | 76.52±10.56 | 0.289 |
|  | 2^nd^ hour | 79.31±14.01 | 77.52±10.80 | 0.479 |
|  | 3^rd^ hour | 79.63±13.14 | 79.58±10.47 | 0.982 |
|  | 4^th^ hour | 78.21±15.55 | 76.96±10.92 | 0.648 |
|  | 5^th^ hour | 78.71±15.37 | 75.73±10.28 | 0.265 |
|  | T3 | 77.10±11.58 | 77.80±9.91 | 0.746 |
| Heart rate (beats/minute) | T1 | 77.74±10.88 | 82.02±11.22 | 0.056 |
|  | T2 | 76.04±10.36 | 80.36±12.67 | 0.065 |
|  | 1^st^ hour | 88.92±11.39 | 90.90±11.68 | 0.393 |
|  | 2^nd^ hour | 92.10±13.32 | 93.20±11.77 | 0.665 |
|  | 3^rd^ hour | 93.06±14.18 | 94.20±12.92 | 0.677 |
|  | 4^th^ hour | 94.40±13.51 | 98.06±15.05 | 0.210 |
|  | 5^th^ hour | 96.04±13.77 | 96.43±13.65 | 0.890 |
|  | T3 | 93.12±14.70 | 95.80±13.15 | 0.339 |
| SpO_2_ (%) | T1 | 98.52±0.97 | 98.12±3.36 | 0.421 |
|  | T2 | 98.64±1.10 | 98.56±0.91 | 0.693 |
|  | 1^st^ hour | 80.86±7.72 | 83.68±5.48 | 0.038^*^ |
|  | 2^nd^ hour | 80.02±5.85 | 82.66±5.11 | 0.019^*^ |
|  | 3^rd^ hour | 82.08±5.42 | 84.86±5.02 | 0.009^*^ |
|  | 4^th^ hour | 82.73±5.97 | 83.10±5.33 | 0.746 |
|  | 5^th^ hour | 81.75±7.11 | 83.08±5.54 | 0.306 |
|  | T3 | 83.10±5.15 | 85.47±5.14 | 0.026^*^ |

The data are presented as the mean ±standard deviation. IH, intermittent hypoxia; SpO_2_, peripheral oxygen saturation. T1: at recruitment, T2: before entering the hypoxic chamber, T3: at the end of the 6-hour hypoxia exposure or when withdrawing. *, p<0.05.

Supplement Table 3. Brain injury and inflammatory indicators between groups

|  | Time points | Groups | | P |
| --- | --- | --- | --- | --- |
|  |  | Control group | IH group |  |
| PGP9.5 (pg/ml) | T1 | 91.55±50.30 | 88.74±32.53 | 0.749 |
|  | T2 | 96.48±77.81 | 89.66±38.63 | 0.594 |
|  | T3 | 120.71±89.74 | 92.63±35.88 | 0.047^*^ |
| GFAP (pg/ml) | T1 | 8.06±2.32 | 8.16±2.87 | 0.855 |
|  | T2 | 9.04±5.49 | 9.23±3.34 | 0.837 |
|  | T3 | 7.72±3.16 | 8.16±3.14 | 0.492 |
| S100β (pg/ml) | T1 | 93.03±56.39 | 102.42±80.99 | 0.533 |
|  | T2 | 118.27±99.89 | 113.46±70.41 | 0.795 |
|  | T3 | 97.12±91.72 | 91.93±55.50 | 0.750 |
| Interleukin 6 (pg/ml) | T1 | 2.00±0.50 | 1.93±0.44 | 0.477 |
|  | T2 | 1.98±0.32 | 1.91±0.44 | 0.374 |
|  | T3 | 2.15±0.67 | 2.12±0.53 | 0.841 |
| CRP (mg/l) | T1 | 0.97±0.99 | 1.33±1.24 | 0.119 |
|  | T2 | 1.27±1.55 | 1.07±0.99 | 0.458 |
|  | T3 | 1.38±1.84 | 1.05±1.09 | 0.290 |

The data are presented as the mean ±standard deviation. IH, intermittent hypoxia; PGP 9.5, protein gene product 9.5; GFAP, glial fibrillary acidic protein; S100β, calcium channel binding protein S100 subunit beta; CRP, C-reactive protein. T1: at recruitment, T2: before entering the hypoxic chamber, T3: at the end of the 6-hour hypoxia exposure or when withdrawing. *, p<0.05.
